# Mucosal Melanomas of the Head and Neck: The Role of Postoperative Radiation Therapy

**DOI:** 10.5402/2012/785131

**Published:** 2012-04-11

**Authors:** Kunal Saigal, Donald T. Weed, Isildinha M. Reis, Arnold M. Markoe, Aaron H. Wolfson, Janet Nguyen-Sperry

**Affiliations:** ^1^Department of Radiation Oncology, Sylvester Comprehensive Cancer Center, University of Miami Miller School of Medicine, University of Miami, 1475 NW 12th Ave, Suite 1500, Miami, FL 33136, USA; ^2^Department of Radiation Oncology, Jackson Memorial Hospital, Miami, FL 33136, USA; ^3^Department of Otolaryngology—Head and Neck Surgery, Sylvester Comprehensive Cancer Center, University of Miami Miller School of Medicine, University of Miami, Miami, FL 33136, USA; ^4^Division of Biostatistics, Department of Epidemiology and Public Health and Biostatistics and Bioinformatics Core, Sylvester Comprehensive Cancer Center, University of Miami Miller School of Medicine, University of Miami, Miami, FL 33136, USA

## Abstract

*Objectives*. Mucosal melanomas are rarer than their cutaneous counterparts and are associated with a poorer prognosis. We report the clinical outcomes of patients with mucosal melanomas of the head and neck region generally treated with definitive surgery followed by postoperative radiation therapy (RT). *Methods*. We reviewed the records of 17 patients treated at the University of Miami in 1990–2007. Patients generally received conventionally fractionated RT regimens to the postoperative bed. Elective nodal RT was not routinely delivered. Eight patients received adjuvant chemotherapy or immunotherapy. *Results*. Median followup was 35.2 months (range 5–225). As the first site of failure: 3 patients recurred locally, 2 regionally and 2 distantly. All 3 patients who recurred locally had not received RT. Of the 5 locoregional recurrences, 4 were salvaged successfully with multimodality therapy with no evidence of disease at last followup. Overall survival was 64.7% at 2 years and 51.5% at 5 years. *Conclusions*. Patients with mucosal melanoma of the head and neck are best treated with surgery to achieve negative margins, followed by postoperative RT to optimize local control. Elective nodal irradiation may not be indicated in all cases, as regional failures were not predominant. Distant metastases were fewer when compared to historical data, potentially due to advancements in adjuvant therapies as well as aggressive multi-modality salvage at time of failure.

## 1. Introduction

Mucosal melanomas are exceedingly rare tumors which comprise a small subset of all melanomas but are associated with an even poorer prognosis than their cutaneous counterparts [1]. While mucosal primaries account for less than 2% of all melanomas in the United States, approximately 50% of mucosal melanomas occurs in the head and neck region [2]. These lesions most commonly present in the oral cavity, nasal cavity, or paranasal sinuses, all of which are lined with ectodermal derived mucosa. Other head and neck sites such as the pharynx and larynx are endodermal in their origin and therefore are rarely affected [3].

Despite their rarity, the aggressive nature of these tumors has been well established, with 5-year overall survival rates generally described as 30% at best [3–5]. The poor prognosis is typically associated with early presentation of distant metastases despite adequate locoregional control [1].

As these lesions are uncommonly encountered, there remains a paucity of data to clearly delineate optimal treatment regimens. Primary treatment has typically consisted of surgery and/or radiation therapy (RT). However, there remain no prospective data to formally compare treatment modalities. Available data generally comes from single-institution retrospective series and often includes patients treated decades ago with older local and systemic modalities.

The role of RT in particular remains unclear. While adjuvant RT has been shown to improve local control in multiple series, an improvement in survival is yet to be observed on a consistent basis [5]. This is likely due to an inherent selection bias, with more advanced disease being treated with aggressive, combined-modality regimens. This issue, along with the competing risk of potentially lethal distant metastases in more advanced disease, makes a survival advantage difficult to demonstrate in a retrospective setting. Furthermore, the rarity of this disease entity limits the statistical power of single-institution series.

We report our results of a modern experience of patients with primary mucosal melanomas of the head and neck treated at the University of Miami from 1990–2007. We seek to generate hypotheses in regards to the optimal treatment paradigm for patients in the contemporary era of advanced radiation delivery techniques, as well as in the setting of newer systemic cytotoxic and immunologic therapies.

## 2. Materials and Methods

We retrospectively reviewed the records of 17 patients with primary mucosal melanomas of the head and neck treated with a curative intent at the University of Miami and affiliated hospitals from 1990 through 2007 in an institutional review board-approved outcomes analysis. Patients were included if they had a previously untreated, newly diagnosed non metastatic primary mucosal melanoma of the head and neck region.

Patient and disease characteristics included age, gender, race, primary site of disease, histologic subtype, disease stage, and treatment received. Although margin status was available in all cases, depth of invasion was generally not reported as tumors are often unable to be resected en-bloc, particularly in sinonasal sites. As no modern staging system exists for mucosal melanomas, we retrospectively staged using the 2009 AJCC staging system for head and neck cancers based upon the primary site of disease [6].

 Overall survival (OS) was defined as the time from surgery/biopsy to death from any cause, with surviving patients censored at date of last followup. Disease freedom (DF) was defined as the elapsed time from date of surgery/biopsy to earliest occurrence of local, regional, or distant failure. Patients who died of unrelated causes were also censored at last followup. Overall survival and disease freedom were estimated using the Kaplan-Meier method [7]. The rates of local, regional, and distant failures were estimated by the method of cumulative incidence (CI) as described by Gray [8, 9]. Statistical analyses were conducted using SAS software version 9.2 (SAS Institute Inc., Cary, North Carolina) and R software version 2.11.1.8. Due to the limited patient numbers in this single-institution retrospective analysis, subset analyses are not reported as they are unlikely to have meaningful results. Instead, we generally report our results using descriptive statistics.

## 3. Results

### 3.1. Patient Characteristics

Of the 17 patients, 10 (59%) were men and 7 (41%) were women. The median age was 66 years (range, 27–84 years). Fourteen (82%) of patients were white and three (18%) were African American. Thirteen (76%) of patients presented with melanoma NOS, while four (24%) had a spindle-cell subtype. Median followup was 35.2 months (range 5–225) overall and 61 months in surviving patients.

The primary site of disease was in the sinonasal cavity for 11 (65%) patients and oral cavity for 6 (35%) patients. Based upon the 2009 AJCC guidelines for head and neck tumors, six (35%) patients were clinically staged as having T1, one (6%) as T2, three (24%) as T3, and five (29%) as T4 disease. One patient's primary tumor could not be adequately staged and assigned a stage of Tx (6%). Fifteen (88%) patients were clinically N0 and two (12%) were N2. All patients presented without evidence of metastatic disease.

### 3.2. Treatment

Sixteen of the 17 patients (94%) underwent surgical resection upfront while one (6%) was treated with definitive RT. Of the 16 patients who underwent upfront surgical resection, surgical margins were negative in 13 (81%) patients and positive in three (19%) patients. Patients who presented with palpable neck disease underwent at minimum an ipsilateral neck dissection. Eleven patients (65%), received postoperative RT to the involved sites of disease. The median radiation dose delivered was 59.4 Gy (range, 54–61.2 Gy), typically in 1.8–2 Gy per daily fraction to the involved sites of disease. Hypofractionated treatment regimens were not used. One patient (6%) however, was treated with a hyperfractionated regimen at 1.2 Gy b.i.d. and one patient (6%) did receive elective nodal RT. Seven patients (41%) received adjuvant immunotherapy while one patient (6%) received adjuvant chemotherapy. Patient and treatment characteristics are summarized in Table 1.

### 3.3. Failures

Recurrence data is based upon the first site of failure. Overall, seven of 17 (41%) patients recurred at present followup. Of these, three patients recurred locally, two regionally, and two distantly. Incidentally, all three patients who recurred locally had not received postoperative RT (Table 2). When patients did recur locally or regionally, they were salvaged aggressively, often with a combination of surgery, RT, and/or systemic therapy. Using this aggressive approach, four of the five locoregional recurrences were salvaged successfully, with no evidence of disease at last followup. The patient who was not salvaged successfully went on to develop distant metastases and died in fewer than six months since time of noted failure. 

**Figure 1:**
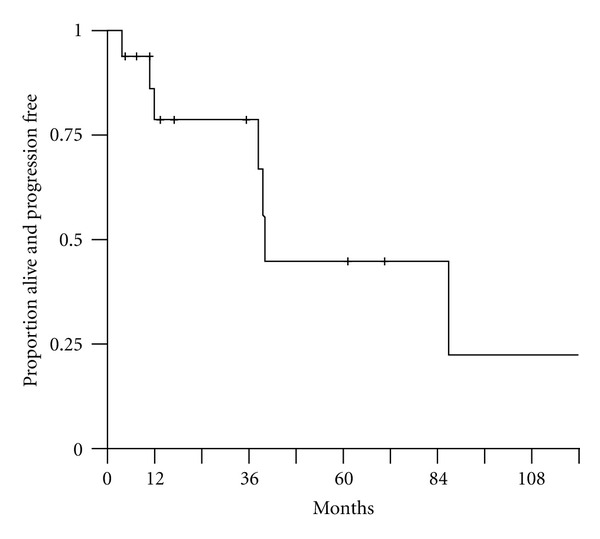
Disease-freedom.

**Figure 2:**
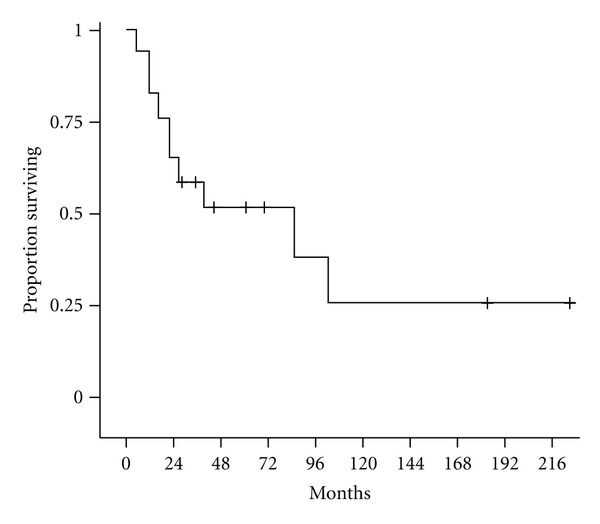
Overall survival.

Based upon cumulative incidence estimates, local control at 2 years was excellent 92% and preserved at 5 years at 81%. Distant control at 2 and 5 years was 85.9% (Table 3). Disease freedom, censoring deaths due to unrelated causes, was 78.1% at 2 years and 44.6% at 5 years (Table 4, Figure 1). Overall survival rates were 64.7% at 2 years and 51.5% at 5 years (Table 4, Figure 2). 

## 4. Discussion

Both historical and modern series demonstrate that mucosal melanomas of the head and neck remain a particularly aggressive malignancy. Due to their propensity to recur distantly in addition to locally, they exhibit even more aggressive behavior than typical head and neck malignancies. The optimal treatment regimen for patients remains unclear as there are no prospective data, nor even a comprehensive staging system, for this rare disease. Depth of invasion, which is a well established element of risk stratification in cutaneous disease, is difficult to evaluate and thus not well studied in mucosal primaries. The lack of clearly defined prognostic indicators makes it difficult to assess which patients may truly benefit from more aggressive treatment regimens. Earlier, primary site of disease was felt to be an important prognostic indicator, with sinonasal primaries having a poorer outcome than lesions originating in the oral cavity [3]. However, this has not been supported consistently by more modern reports, which suggest that the survival is similar for all head and neck primary sites [5, 10].

### 4.1. Surgery and Posoperative Radiation Therapy

Wide surgical resection has become the mainstay of initial therapy when feasible [11]. However, the types of en-bloc tumor resections which allow wide surgical margins typically employed in cutaneous melanoma are often not feasible in head and neck mucosal primaries, due to their proximity to critical normal structures. While advances in surgical technique have enabled more aggressive local resections in large part due to the advent of more sophisticated reconstruction techniques such as microvascular-free tissue transfer, these techniques still do not allow the uniform application of wide (1.5 to 2 cm) resection margins in these locations.

Postoperative RT has shown to improve local control in several retrospective series [5, 10]. Whether this improvement translates to an improvement in prognosis remains unclear however, as only one report has demonstrated an overall survival benefit with the use of postoperative RT [12].

Improved local control likely corresponds with improved outcomes, as was seen in the Mandolis meta-analysis in which rates of distant metastasis were 73.1% in patients with local failure while 52.1% in those with local control [3]. This series suggested that local failure portends distant failure, which is often lethal. Individual series, however, have not consistently shown this relationship between local and distant control [13].

The inability of individual series to demonstrate significant overall advantages with the addition of RT is skewed by the inherent selection bias each retrospective analysis, in which patients with more extensive disease were often treated more aggressively. Postoperative RT may therefore potentially benefit patients, despite retrospective series paradoxically reporting better outcomes in patients treated with surgery alone. Our data suggests that the addition of conventionally delivered RT leads to improved local control, as all three patients who recurred locally had not received postoperative RT to the tumor bed.

### 4.2. Elective Nodal Irradiation

While it appears that RT to the postoperative bed improved local control, the potential benefit of regional RT is less clear. In a series from the University of Florida, surgery followed by postoperative RT led to improved local control versus definitive RT. Additionally, six patients received elective nodal RT to clinically uninvolved cervical nodal regions. A trend towards improvement in regional control was observed with the addition of neck irradiation at 2.5 years.

The authors of this University of Florida series agree that surgery with postoperative RT should be used in nearly all cases of head and neck mucosal melanomas. However, they also suggest that elective nodal irradiation should be used to address subclinical regional disease. Incidentally, this recommendation is based upon very small patient numbers, with regional control being achieved in 5/6 patients receiving elective neck RT, versus 6/8 patients not receiving elective nodal therapy [14].

In our series, 16/17 patients did not receive elective nodal RT. Only two patients failed regionally and were successfully salvaged with long-term control with aggressive therapy. Therefore, we do not presently recommend routine elective nodal irradiation of the neck, as the benefits may not outweigh its morbidity in all cases. Patients with oral cavity primaries have a higher rate of cervical nodal metastasis and should be evaluated on a case-by-case basis, with consideration of elective nodal irradiation based upon tumor size, location, and so forth. This conclusion suggested by our data is in accord with the current NCCN practice guidelines for this disease entity [15]. Sentinel lymph node biopsy, which has become a mainstay in cutaneous melanoma and is showing promise in head and neck squamous cell carcinomas, may be of particular value in elucidating this treatment dilemma [16].

### 4.3. Definitive Radiation Therapy

Due to the location and extent of these tumors in the head and neck region, a wide surgical resection is sometimes not possible without leaving the patient with significant functional or cosmetic deficits. For these reasons, definitive RT has been considered for mucosal melanomas of the head and neck.

While initial response rates are reasonable, long-term outcomes with photon irradiation have generally been poor. This is often due to development of distant metastases despite local disease control [17]. Due to the potential radioresistance of melanoma cells, hypofractionated regimens and dose-escalation have been adopted order to more effectively eradicate the primary tumor. These regimens must be used with caution in head and neck sites, however, to avoid significant late toxicities of RT. Modern RT delivery however, including stereotactic techniques, may allow for dose escalation and/or hypofractionation, while preferentially sparing surrounding normal tissues.

 Higher linear energy transfer RT has also been considered in this setting. In addition to the physical advantage of greater energy deposition per unit length, proton and heavy ion beams also have a spatial selectivity due to their finite range and depth-dose distribution [18]. This dose deposition profile is of particular interest in the head and neck region, where critical normal structures often lie adjacent to the tumor itself.

 In a recent report from Japan, carbon ion beams were used as definitive therapy for previously untreated mucosal melanomas of the head and neck. Five-year local control rate for patients was 84.1%, while five-year overall survival was estimated at 27%, with minimal severe long-term toxicities. Distant metastases occurred in 56% of patients overall, however, corresponding to the overall survival described above. However, 34/40 patients in this series who developed distant metastases were without local recurrence, which depicts the efficacy of this therapy in terms of local control [19].

The rate of local control described the Japanese series with radiation alone is at par with most surgical series, while overall survival is similar [3, 5, 10]. Given the potential morbidities of surgery in this disease, definitive RT with heavy ions should be considered as a noninvasive approach in patients unable to undergo surgery. Unfortunately, carbon ion therapy is of limited use in the postoperative setting, as the increased biological effect can be deleterious to normal tissues.

### 4.4. Systemic Therapies

While local and regional control may be improved with advancements in surgical and radiation delivery techniques, the poor prognosis of this rare disease centers upon the rate of distant metastases. The early presentation of distant disease, even with adequate local control, suggests the need for early intervention with systemic therapies.

 Initial results with traditional chemotherapeutic agents in both cutaneous and mucosal variants of melanoma have been disappointing [20]. This has led to a movement towards immunotherapies in melanoma and other resistant tumors, in an effort to modulate the patient's own immune system to respond to the tumor cells as a foreign entity [3]. These therapies could potentially be delivered concurrently with RT to further enhance tumor cell damage.

 In the current series, seven of 17 patients received immunotherapy in the adjuvant setting, while one additional patient was treated in the salvage setting. Our improvement in 5-year survival compared to historical series may be in part due to the early use of systemic therapies which may have potentially prevented distant metastatic disease from presenting or addressed subclinical disease prior to it becoming apparent. Our data also shows that while local and regional failures can be successfully salvaged, it remains difficult to provide disease control once distant metastases are present. A detailed analysis of systemic therapies is certainly beyond the scope of this paper, but our results are hypothesis generating in terms of the benefit of early adjuvant systemic therapies.

### 4.5. Limitations

This analysis is clearly limited by its retrospective nature, small patient numbers, and lack of formal toxicity data with grading for all patients. It is unique, however, in terms of its modern treatment techniques and use of systemic therapies. While the patient number may appear small, it represents the patient volume of a single institution with an active head and neck cancer program and is similar to other analyses that included patients treated over longer time frames [3, 14]. Although we do not have graded toxicity data, severe functional toxicities such as blindness or damage to the brainstem were not noted in any patients. This is as expected since patients were generally treated with 3D-conformal or intensity modulated radiation therapy (IMRT).

## 5. Conclusions

Mucosal melanomas of the head and neck remain a rare disease entity with an aggressive natural history and poor long-term prognosis. The mainstay of therapy remains definitive surgery followed by postoperative RT to the tumor bed. This aggressive multimodality approach appears to improve local control, even in patients with advanced disease at presentation. This improvement in local control has not necessarily correlated with improved overall outcomes in individual retrospective series, however, due to limitations of each series. Given the lower rates of cervical nodal metastases, particularly in the case of sinonasal disease, as well as the potential for long-term control after successful salvage therapy at the time of neck failure, we currently do not recommend the routine use of elective neck irradiation. Heavy-ion therapy, although not widely available, has been shown to provide similar control rates to surgery followed by photon irradiation. It should be considered particularly in patients who have unresectable disease or are unsuitable for surgery. Our improvement in overall survival rates compared to historical series may reflect improvements in delivery of local therapies in the modern era, as well as the advent of systemic therapies which prevent locoregional recurrence and distant dissemination of disease.

##  Disclosure

The authors have no financial support or other disclosures to report. Part of it presented at American Society for Radiation Oncology (ASTRO) 52nd Annual Meeting, November 2, 2010. San Diego, CA, USA.

##  Conflict of Interests

The authors have no conflict of interests to report.

## Figures and Tables

**Table 1:** Patient and treatment characteristics. Patients were staged retrospectively using the 2009 AJCC cancers staging system. Note that most patients were treated in the postoperative setting with conventionally fractionated radiation therapy.

Patient	Age	Sex	Site	Histologic subtype	T	N	M	Surgery	Margins	XRT	XRT Dose (Gy)	XRT fractionation	Immuno	Chemo
1	57	Female	Sinonasal	Mucosal melanoma, NOS	T1	N0	M0	Yes	Negative	Yes	58.9	1.2 b.i.d.	Yes	No
2	65	Male	Oral cavity	Mucosal melanoma, NOS	T1	N0	M0	Yes	Negative	Yes	60	2 daily	No	No
3	64	Male	Oral cavity	Mucosal melanoma, NOS	T4	N0	M0	Yes	Negative	No			No	No
4	27	Male	Sinonasal	Mucosal melanoma, NOS	T4	N0	M0	Yes	Negative	Yes	59.4	1.8 daily	No	No
5	61	Female	Sinonasal	Mucosal melanoma, NOS	T4	N0	M0	Yes	Negative	Yes	59.4	1.8 daily	Yes	No
6	77	Female	Sinonasal	Mucosal melanoma, NOS	T3	N0	M0	Yes	Positive	Yes	59.4	1.8 daily	Yes	No
7	46	Female	Sinonasal	Mucosal melanoma, NOS	T1	N0	M0	Yes	Negative	Yes	unk		Yes	No
8	62	Female	Sinonasal	Spindle cell melanoma	T3	N0	M0	Yes	Negative	Yes	unk		Yes	No
9	76	Female	Sinonasal	Mucosal melanoma, NOS	T1	N0	M0	Yes	Negative	Yes	unk		No	No
10	65	Male	Oral cavity	Mucosal melanoma, NOS	T1	N2b	M0	Yes	Negative	Yes	62	2 daily	Yes	No
11	68	Male	Sinonasal	Mucosal melanoma, NOS	T2	N0	M0	Yes	Negative	No			No	No
12	82	Male	oral cavity	Spindle cell melanoma	Tx	N0	M0	Yes	Negative	No			No	No
13	74	Female	Sinonasal	Spindle cell melanoma	T3	N0	M0	No	N/A	Yes	61.2	1.8 daily	No	Yes
14	83	Female	Sinonasal	Mucosal melanoma, NOS	T3	N0	M0	Yes	Negative	Yes	54	1.8 daily	Yes	No
15	51	Female	Oral cavity	mucosal melanoma, NOS	T4	N2a	M0	Yes	Positive	Yes	60	2 daily	No	No
16	77	Male	Sinonasal	Mucosal melanoma, NOS	T4	N0	M0	Yes	Positive	No			No	No
17	66	Female	Oral cavity	Spindle cell melanoma	T1	N0	M0	Yes	Negative	No			No	No

**Table 2:** Recurrences. Note that all 3 local failures occurred in patients who did not receive postoperative radiation therapy to the tumor bed.

Recurrence	*n*	Percentage
Yes	7	41
No	10	59
Site of recurrence		

Local	3	17
Regional	2	12
Distant	2	12

**Table 3:** Rates of local, regional, and distant control as estimated by cumulative incidence.

Time (months)	Local Control	Locoregional Control	Distant Control
24	92.2% (69.2–99.6%)	92.2% (69.2–99.6)	85.9% (62.7–98%)
60	81.0% (51.2–97.9%)	58.7% (29.4–89.4%)	85.9% (62.7–98%)

**Table 4:** Kaplan Meier estimates of disease-freedom and overall survival.

Time (months)	Disease freedom	Overall survival
24	78.1% (56–92.5%)	64.7% (37.7–82.3%)
60	44.6% (14.6–71.3%)	51.5% (25.7–72.3%)
